# Kinematic correlates of early speech motor changes in cognitively intact APOE-ε4 carriers: a preliminary study using a color-word interference task

**DOI:** 10.3389/fneur.2025.1649729

**Published:** 2025-12-17

**Authors:** Mehrdad Dadgostar, Lindsay C. Hanford, Jordan R. Green, Brian D. Richburg, Averi Taylor Cannon, Nelson V. Barnett, David H. Salat, Steven E. Arnold, Marziye Eshghi

**Affiliations:** 1 MGH Institute of Health Professions, Charlestown, MA, United States; 2 Athinoula A. Martinos Center for Biomedical Imaging, Harvard-MIT Health Sciences and Technology, Charlestown, MA, United States; 3 Department of Psychology, Harvard University, Cambridge, MA, United States; 4 Speech and Hearing Bioscience and Technology, Harvard University, Boston, MA, United States; 5 Department of Radiology, Massachusetts General Hospital and Harvard Medical School, Boston, MA, United States; 6 Neuroimaging Research for Veterans Center, VA Boston Healthcare System, Boston, MA, United States; 7 Department of Neurology, Massachusetts General Hospital and Harvard Medical School, Boston, MA, United States

**Keywords:** APOE-ε4 genotype, genetic risk factor, Alzheimer’s disease, kinematics, stroop-induced speech tasks, color-word interference, speech biomarkers

## Abstract

**Introduction:**

Alzheimer’s disease (AD) is the most prevalent form of dementia and a major public health challenge. In the absence of a cure, accurate and innovative early diagnostic methods are essential for proactive life and healthcare planning. Speech metrics have shown promising potential for identifying individuals with mild cognitive impairment (MCI) and AD, prompting investigation into whether speech motor features can detect elevated risk even prior to cognitive decline. This preliminary study examined whether speech kinematic features measured during a color-word interference task could distinguish cognitively normal APOE-ε4 carriers (ε4^+^) from non-carriers (ε4^−^).

**Methods:**

Sixteen cognitively normal older adults (*n* = 9 ε4^+^, *n* = 7 ε4^−^) completed a sentence-based color–word interference task while three-dimensional electromagnetic articulography recorded lower-lip movements. Lip movement duration (s), average speed (mm/s), and range of movement (mm³) were extracted for three sentence segments: pre-interference, during-interference, and post-interference. Difference measures (ΔDuring–Pre, ΔDuring–Post) were computed to quantify task-related modulation. Descriptive statistics and independent t-tests were used to examine group-level trends. For classification, a support vector machine (SVM) with a degree-2 polynomial kernel and leave-one-out cross-validation evaluated all feature combinations derived from the 15 kinematic measures.

**Results:**

Although no group differences reached statistical significance after accounting for multiple testing, several features showed moderate effect sizes. The optimal SVM model achieved 87.5% cross-validated accuracy (precision 88.9%, sensitivity 88.9%, specificity 85.7%) using three features: (1) lip movement duration during the pre-interference segment, (2) average lip speed during interference, and (3) the change in lip movement range from pre- to during-interference segments (ΔDuring–Pre).

**Discussion:**

These findings suggest that lip kinematic responses to mild cognitive–motor interference may capture subtle neuromotor differences associated with APOE-ε4 status in cognitively intact older adults. The identified features point to potential alterations in anticipatory motor planning, interference susceptibility, and articulatory adaptability in ε4^+^ individuals. However, the small sample size, risk of overfitting, and sex imbalance limit interpretability. Thus, these results should be viewed as hypothesis-generating. Larger, sex-balanced, and longitudinal studies are needed to validate these candidate markers and clarify their role in multimodal early AD risk stratification.

## Introduction

Apolipoprotein E4 (APOE-ε4) is the major genetic risk factor for Alzheimer’s disease (AD), its presence significantly increasing the likelihood of developing the condition (1). The presence of one APOE-ε4 allele raises the risk of AD by three to four times, while homozygous carriers face a 12- to 15-fold increase (2–5). Moreover, the APOE-ε4 allele exerts a profound influence on cognitive processes and motor control, particularly in the context of aging and neurodegenerative diseases (6–9).

APOE-ε4 status has been associated with accelerated decline in memory, executive function, processing speed, attention, and spatial cognition (7, 10–12). These deficits are linked to structural and functional brain changes, including reduced hippocampal volume, disrupted brain connectivity, and increased vulnerability to neurodegenerative processes (13–17). The allele’s role in mild cognitive impairment (MCI) further underscores its clinical relevance. APOE-ε4 status can increase susceptibility to MCI, particularly the amnestic subtype, and is associated with faster progression from MCI to AD (18–21). In addition, APOE-ε4 carriers experience more pronounced decline in motor abilities compared to non-carriers, which can manifest as decreased muscle strength, impaired coordination, and a greater risk of falls (6, 22–25). The effects on motor function are attributed to apolipoprotein’s role in neuroplasticity, neuromuscular junction integrity, and overall brain health (26–29). Gait speed, an important marker of physical function, declines more rapidly in APOE-ε4 carriers, emphasizing the allele’s contribution to physical frailty in aging populations (8, 23, 25).

The Stroop task, a widely used neuropsychological test, effectively assesses cognitive control, particularly selective attention and inhibitory control (30, 31). The task requires individuals to suppress automatic responses, such as reading a word, in favor of identifying the ink color in which the word is printed, creating a conflict between the two responses. Individuals with MCI and AD consistently perform worse on Stroop tasks compared to cognitively healthy older adults (32–37), exhibiting longer reaction times and greater error rates, particularly on incongruent trials where word-color and semantic meaning differ (33, 38). These deficits are thought to reflect impairments in executive functions, including reduced inhibitory control and slower processing speed, both hallmark cognitive deficits in MCI and AD. Furthermore, Stroop task performance has been shown to correlate with underlying AD biomarkers, such as increased amyloid-*β* and tau pathology, making it a valuable tool for early detection (39–41).

Variants of the Stroop task, including the oral Stroop task, introduce a verbal component, making it particularly relevant for studying speech motor control under cognitive load. In individuals with MCI and AD, the combination of cognitive conflict and the motor demands of speech can exacerbate performance deficits, leading to slower response times, increased speech hesitations, and more frequent articulation errors (33, 42). These findings suggest that Stroop-inspired tasks can effectively probe the cognitive-motor interface, particularly in populations at risk for neurodegenerative diseases.

Speech alterations have long been recognized as a hallmark of AD, with prior studies linking AD-related neuropathology to changes in prosody, fluency, acoustics, and linguistic skills (43–46). These disruptions have made speech an attractive target for early diagnosis, particularly through acoustic analyses and self-reported communication difficulties. However, existing diagnostic approaches often rely on global acoustic features or subjective reports, which may lack the resolution needed to detect subtle neuromotor disruptions that precede overt cognitive decline. The long-term goal of this research is to advance speech as a sensitive and scalable biomarker for preclinical AD with the capability to capture early, subtle motor signatures before clinical symptoms emerge. Building on this motivation, the current study introduces the application of speech kinematic analysis to investigate cognitive-motor interactions in individuals genetically predisposed to AD. The primary aim of this preliminary study is to determine whether lip kinematic features recorded under minimal cognitive load can accurately classify individuals based on APOE-ε4 status (ε4^+^ vs. ε4^−^). By focusing on subtle motor signatures during low-demand speech tasks, this approach seeks to identify early, preclinical markers of AD risk that are scalable and non-invasive. We hypothesized that individuals with APOE-ε4 status would demonstrate early disruptions in speech motor performance compared to noncarriers.

Prior work from our group demonstrated that cognitively intact APOE-ε4 carriers exhibit early neuromuscular alterations in orofacial speech production, detectable via surface electromyography (EMG). While EMG effectively captures muscle activation patterns, it lacks the spatial resolution to fully characterize articulatory trajectories and inter-articulator coordination. Kinematic analysis, by contrast, enables precise quantification of the spatial and temporal dynamics of articulator motion. This approach has already shown diagnostic utility in detecting early bulbar dysfunction in neurodegenerative diseases such as amyotrophic lateral sclerosis (ALS) (47–50), where kinematic markers have demonstrated greater sensitivity to early neuromotor decline than either acoustic measures or subjective reporting.

To probe these dynamics in the context of preclinical AD, we employed a modified color-word interference speech task designed to introduce mild cognitive conflict while preserving naturalistic speech production. Unlike conventional Stroop paradigms that rely on isolated words or short utterances, our task embeds cognitive interference within full sentences, maintaining linguistic continuity while subtly taxing cognitive control. This dual approach (i.e., leveraging the fine-grained sensitivity of lip kinematics and introducing controlled cognitive load) was designed to reveal early neuromotor vulnerability in ε4 carriers who remain clinically asymptomatic. These innovations aim to overcome the limitations of traditional speech-based assessments and move the field toward more precise, high-resolution biomarkers of early AD risk. This design allows for the assessment of articulatory coordination as participants navigate mild cognitive conflict, offering a more ecologically valid and targeted window into the interplay between cognitive control and speech motor function.

## Methods

### Participants

The participants consisted of 16 older adults ranging in age from 50–90 years. Data was collected at the Speech Physiology and Neurobiology of Aging and Dementia (SPaN-AD) Lab as part of an ongoing longitudinal study. All participants in the study held an education level of at least 16 years. All participants were native speakers of American English and exhibited normal cognition as assessed by a neuropsychologist. The cognitive status of the participants was examined by a comprehensive array of standard neuropsychological tests that assess attention, processing speed, learning, memory, language, and executive function. Details of the neuropsychological assessments are provided by Eshghi et al. (51). In addition, all participants scored 0 on the Clinical Dementia Rating (CDR) scale global score and less than 11 on the Geriatric Depression Scale (GDS). All participants exhibited normal, age-appropriate hearing status in at least one ear as verified by a pure tone audiometer (Earscan 3, Micro Audiometric Corp.) and normal visual acuity (52). None of the participants showed a clinical diagnosis of MCI, any neurological condition, or were using any psychoactive drugs. Participants were genotyped with respect to the APOE-ɛ4 gene and were put into two groups: (1) carriers of APOE-ɛ4 who possess heterozygous ɛ3–ɛ4 alleles or homozygous ɛ4–ɛ4 alleles and (2) noncarriers of APOE-ɛ4 who possess homozygous ɛ3–ɛ3 alleles. Characteristics of the participants in each group are provided in Table 1. The study was reviewed and approved by the MGB Institutional Review Board.

**Table 1:** Baseline characteristics stratified by genotype carrier status.

Variables	All participants	ɛ4^−^	ɛ4^+^	
	(*n* = 16)	(*n* = 7)	(*n* = 9)	*p*-value
Female, *n* (%)	8 (50.00)	2 (28.57)	6 (66.67)	0.057^a^
Family history of AD, *n* (%)	6 (37.50)	2 (28.57)	4 (44.44)	0.515^a^
Age, mean (SD)	67.44 (7.98)	69.86 (9.91)	65.56 (6.06)	0.337^b^
Total years of education, mean (SD)	15.81 (1.97)	15.86 (1.68)	15.78 (2.28)	0.937^b^
MMSE, mean (SD)	29.50 (0.89)	29.57 (1.13)	29.44 (0.73)	0.801^b^
MoCA, mean (SD)	27.81 (2.14)	28.43 (1.51)	27.33 (2.50)	0.297^b^

MMSE, Mini-Mental State Examination; MoCA, Montreal Cognitive Assessment; SD, standard deviation.

a Chi-square test.

b *t*-test.

### Procedure

Lower lip movements were recorded during sentence production using three-dimensional electromagnetic articulography (EMA) (Wave; Northern Digital, Inc.). The EMA system tracked a small (2 mm) electromagnetic sensor affixed to the midline of the lower lip’s vermilion border, using a sampling rate of 100 samples per second. Participants performed color-word interference speech tasks while seated in a supported chair. Stimuli were displayed on a wall-mounted screen positioned approximately six feet in front of them. Audio recordings were obtained using a Williams Sound MIC094 head-mounted microphone positioned approximately 5 cm from the left corner of the lips. The signal was routed through a Focusrite Scarlett 2i2 audio interface and digitized at a sampling rate of 10,000 Hz. Audio and EMA kinematic data were recorded synchronously to ensure precise temporal alignment. Participants were instructed to read sentences at their natural speaking rate and loudness.

### Stimuli

The speech samples consisted of a block of four sentences containing color words, with the font color of each word being incongruent with its semantic meaning (see Table 2). All sentences had comparable syntactic structures, each consisting of 14 words and 18 syllables. To align with the study’s focus on lip movement properties, the sentences contained a high proportion of bilabial consonants.

**Table 2:** Speech stimuli used in the study.

Color-word interference condition		
Pre-interference	During-interference	Post-interference
Pammy and Bobby picked	Red, teal, brown, blue, and pink	Poppies with their mommy
Piper and Mimi bought	Brown, pink, green, teal, and red	Backpacks from my papa
Bebe and Pippa pitched	Green, blue, teal, pink, and brown	Beanbags to the batboy
Barbie and Mabel made	Blue, red, pink, teal, and brown	Bloomers for a baby

Participants were presented with eight blocks of color-word interference sentences. Within each block, the same four sentences were presented in a randomized order. The font color for each color word was also randomly changed across blocks. However, the font color never matched the semantic meaning of the written word in any of the sentences. Two of the four sentences were designated for analysis, while the remaining two served as foil sentences. Data from these foil sentences were collected but not included in the final analyses. Foil sentences were included to reduce predictability and prevent participants from anticipating target sentences, thereby promoting natural speech patterns and improving the reliability of kinematic and acoustic measurements. The speech stimuli were adapted from MacPherson (53), with adjustments made to align with the objectives of the current study.

Participants were instructed to read each sentence while disregarding the conflicting font color, requiring them to suppress the influence of incongruent visual information. This aspect of the task imposed a heightened cognitive load compared to non-interfered parts of the sentences. The increased cognitive demand specifically engaged key cognitive domains, including inhibition (overcoming the automatic tendency to process the font color), selective attention (focusing on the written word while filtering out irrelevant visual distractions), and working memory (maintaining task rules and resolving conflicts between competing stimuli), all of which are susceptible to impairment in MCI and AD (54, 55).

### Kinematic data

SMASH software (56) was used to extract lip kinematic features from the three-dimensional lip movement time series recorded during the production of the entire target sentences. Lip movement duration (s), lip average speed of movement (mm/s), and lip range of movement (mm^3^) were calculated from three distinct sentence segments: the segment preceding the color-interference task (pre-interference), the segment corresponding to the color-interference task (during-interference), and the segment following the color-interference task (post-interference). By assessing kinematic measures of lip movements during these segments, we aimed to gain insights into the anticipatory effects (pre-task), direct effects (during task), and subsequent effects (post-task) of the heightened cognitive load imposed on the motor system.

Additionally, difference measures, including Δ-movement duration, Δ-average speed, and Δ-range of movement, were calculated to assess the impact of the interference task. Difference measures were computed by subtracting the kinematic values of the during-interference segment (i.e., the segment containing color interference) from those of the pre- and post-interference segments. These measures are hereafter referred to as ∆_During-Pre_ and ∆_During-Post_. These measures provide valuable insights into how individual speakers adjust their motor planning and execution in response to the varying cognitive demands of each sentence segment.

### Statistical analyses

Descriptive statistics were computed for each speech kinematic feature within sentence segments. Independent *t*-tests were performed to assess group differences. To adjust for multiple comparisons across 15 features, a Bonferroni-corrected threshold (adjusted *p*-value = 0.003) was applied to control the family-wise type I error rate. In addition, to quantify the magnitude and direction of these differences, standardized effect sizes were calculated using both Cohen’s *d* and Hedges’ *g*, with the latter adjusting for potential small sample bias to provide a more accurate estimate. Although the primary objective was to classify APOE-ε4 carriers (ε4^+^) and non-carriers (ε4^−^) using machine learning models trained on kinematic speech features, we also report descriptive and inferential statistics to enhance interpretability and contextual insight into group-level patterns. To evaluate the adequacy of our sample size for detecting group-level differences in kinematic speech features between APOE-ε4 carriers and non-carriers, we conducted *post hoc* power analyses using the observed effect sizes (Cohen’s *d*) for each feature comparison. For each analysis, the target statistical power was set to 0.80, and the alpha level was set to 0.05, corresponding to conventional thresholds for reliable detection of true effects. These post hoc estimates provide an approximate indication of the sample size required to detect significant differences observed in this study, thereby informing the design of future, adequately powered investigations and enhancing methodological transparency. Given the limited sample, findings from the machine learning classification are reported as exploratory and hypothesis-generating, highlighting potential group-level patterns that merit independent validation in larger cohorts. All statistical analyses were performed in MATLAB (Version 24.2, R2024b; The MathWorks Inc., Natick, MA, United States).

### Classification

The purpose of this study was to identify lip kinematic features recorded during color-word interference speech tasks that can accurately classify APOE-ε4^+^ and APOE-ε4^−^ individuals. Three sets of different features were extracted to capture the dynamic characteristics of lip movement across task conditions. Specifically, lip movement duration, average speed of movement, and range of movement features were calculated (1) across the pre-, during-, and post-interference segments of the color-interference task, (2) on difference values from segments preceding the interference task (∆_During-Pre_), and (3) on difference values from segments following the interference task (∆_During-Post_), yielding a total of 15 features. These 15 features are listed in Table 3. Classification of APOE-ε4 carriers (ε4^+^) versus non-carriers (ε4^−^) was performed using a support vector machine (SVM) classifier with a degree-2 polynomial kernel. An exhaustive search across all 32,767 possible combinations of 15 extracted speech kinematic features was systematically conducted, with each combination evaluated using leave-one-out cross-validation. Feature sets were ranked according to overall classification accuracy, and the optimal set comprised: (1) lip movement duration during the pre-interference segment, (2) average lip speed during the interference segment, and (3) the change in lip movement range from pre- to during-interference segments. This specific combination was selected solely on the basis of its superior performance in the exhaustive search, ensuring robust and discriminative representation of task-related variation. No further model tuning or subjective filtering was performed, and all analytical procedures were implemented in MATLAB (Version 24.2, R2024b; The MathWorks Inc., Natick, MA, United States).

**Table 3:** Mean (SD) values for each feature in ε4^+^ and ε4^−^ groups.

	Genotype: ε4^+^		
Feature	Pre-interference	During-interference	Post-interference
Lip movement duration (s)	1.341 (0.147)	2.156 (0.167)	1.420 (0.157)
Lip average speed of movement (mm/s)	50.014 (14.850)	33.93 (6.613)	47.536 (12.224)
Lip range of movement (mm^3^)	11.600 (1.939)	11.396 (1.509)	11.210 (2.149)

	Genotype: ε4^−^		
Feature	Pre-interference	During-interference	Post-interference
Lip movement duration (s)	1.435 (0.184)	2.274 (0.607)	1.503 (0.167)
Lip average speed of movement (mm/s)	48.618 (11.292)	35.959 (14.247)	46.075 (10.903)
Lip range of movement (mm^3^)	10.325 (1.481)	11.110 (3.798)	11.569 (3.109)

	Genotype: ε4^+^	
Feature	∆_During-Pre_	∆_During-Post_
Lip movement duration (s)	0.821 (0.130)	0.799 (0.190)
Lip average speed of movement (mm/s)	−16.084 (12.956)	−13.606 (12.341)
Lip range of movement (mm^3^)	−0.205 (1.119)	0.186 (1.251)

	Genotype: ε4^−^	
Feature	∆_During-Pre_	∆_During-Post_
Lip movement duration (s)	0.839 (0.546)	0.771 (0.566)
Lip average speed of movement (mm/s)	−12.659 (14.464)	−10.116 (8.045)
Lip range of movement (mm^3^)	0.609 (2.784)	−0.459 (1.112)

## Results

**Figure 1:**
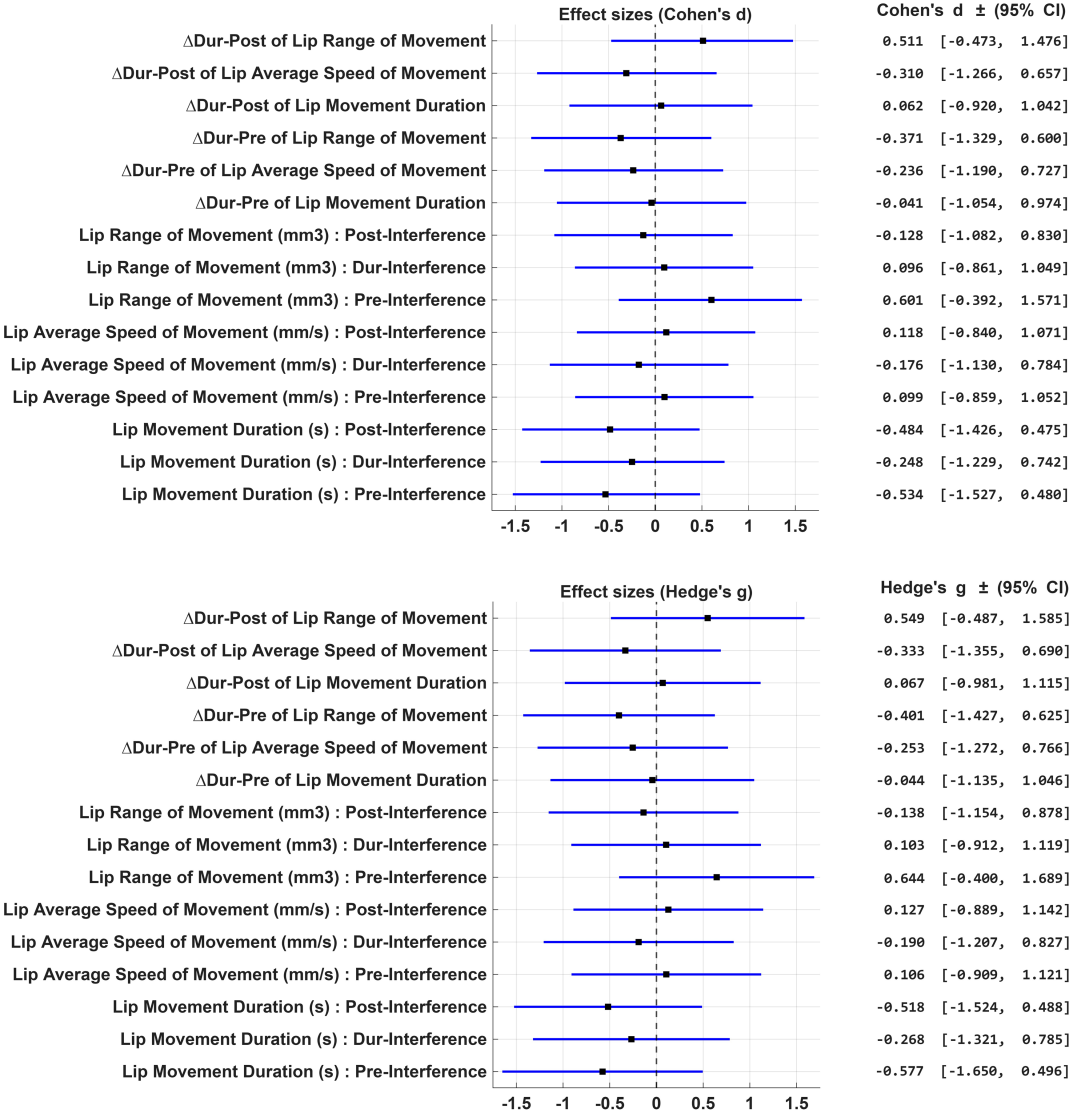
Forest plots show effect sizes for lip movement measures. The top plot uses Cohen’s d, and the bottom plot uses Hedge’s g, with a similar range. Both display confidence intervals for various movements and conditions.

Descriptive statistics for each lip kinematic feature across sentence segments are reported in Table 3. Group comparisons between APOE-ε4 carriers (ε4^+^) and non-carriers (ε4^−^) are summarized in Table 4, including independent t-test *p*-values, Cohen’s *d* and Hedges’ *g* effect sizes, and 95% confidence intervals. Forest plots (Figure 1) visually illustrate the magnitude and direction of these group-level effects for each feature, with positive values indicating higher means in the ε4^+^ group. This visualization facilitates comparison of the discriminatory power of individual kinematic features, supporting interpretation of which metrics most robustly differentiate carriers from non-carriers.

**Table 4:** Statistical comparison of lip kinematic features between ε4^+^ and ε4^−^ groups with effect sizes and 95% confidence intervals.

		Effect size estimate					
Feature		Hedges’ *g*			Cohen’s *d*		
	*t*-test (*p*-value)	Effect size	Confidence intervals		Effect size	Confidence intervals	
			Lower	Upper		Lower	Upper
Lip movement duration (s): pre-interference	0.308	−0.577	−1.650	0.496	−0.534	−1.527	0.480
Lip movement duration (s): during-interference	0.635	−0.268	−1.321	0.785	−0.248	−1.229	0.742
Lip movement duration (s): post-interference	0.333	−0.518	−1.524	0.488	−0.484	−1.426	0.475
Lip average speed of movement (mm/s): pre-interference	0.840	0.106	−0.909	1.121	0.099	−0.859	1.052
Lip average speed of movement (mm/s): during-interference	0.738	−0.190	−1.207	0.827	−0.176	−1.130	0.784
Lip average speed of movement (mm/s): post-interference	0.811	0.127	−0.889	1.142	0.118	−0.840	1.071
Lip range of movement (mm^3^): pre-interference	0.230	0.644	−0.400	1.689	0.601	−0.392	1.571
Lip range of movement (mm^3^): during-interference	0.857	0.103	−0.912	1.119	0.096	−0.861	1.049
Lip range of movement (mm^3^): post-interference	0.802	−0.138	−1.154	0.878	−0.128	−1.082	0.830
∆_During-Pre_ of lip movement duration (s)	0.935	−0.044	−1.135	1.046	−0.041	−1.054	0.974
∆_During-Pre_ of lip average speed of movement (mm/s)	0.640	−0.253	−1.272	0.766	−0.236	−1.190	0.727
∆_During-Pre_ of lip range of movement (mm^3^)	0.490	−0.401	−1.427	0.625	−0.371	−1.329	0.600
∆_During-Post_ of lip movement duration (s)	0.905	0.067	−0.981	1.115	0.062	−0.920	1.042
∆_During-Post_ of lip average speed of movement (mm/s)	0.524	−0.333	−1.355	0.690	−0.310	−1.266	0.657
∆_During-Post_ of lip range of movement (mm^3^)	0.309	0.549	−0.487	1.585	0.511	−0.473	1.476

While none of the group differences reached statistical significance, likely reflecting limited statistical power in the present sample, several kinematic speech features exhibited moderate effect sizes, suggesting potential trends that warrant further investigation in larger cohorts. For instance, lip range of movement (mm^3^) during the pre-interference segment demonstrated a moderate effect size (Cohen’s *d* = 0.60; Hedges’ *g* = 0.64), with *post hoc* power analysis indicating that samples of 45 per group (Cohen’s *d*) or 39 per group (Hedges’ *g*) would be required to achieve 80% power at *α* = 0.05 for detecting significant group differences.

Similarly, the difference in lip range of movement (Δ_Dur-Post_) yielded effect sizes of Cohen’s *d* = 0.51 and Hedges’ *g* = 0.55, corresponding to estimated sample sizes of 62 and 60 participants per group, respectively. Lip movement duration during the post-interference segment also approached this range (Cohen’s *d* = −0.48; Hedges’ *g* = −0.52), with required sample sizes of 68 and 60 per group, respectively, to achieve adequate power.

These analyses underscore that while the present study was underpowered, several candidate features are likely to yield reliable group differences given modest increases in sample size. In particular, targeting approximately 40 participants per group would provide sufficient power to detect effects observed for lip range of movement during the pre-interference segment, while sample sizes of 60–70 per group would be adequate for other features demonstrating comparable effect sizes. These findings provide critical guidance for the design of future studies aiming to robustly detect group-level differences in speech kinematic measures.

**Figure 2:**
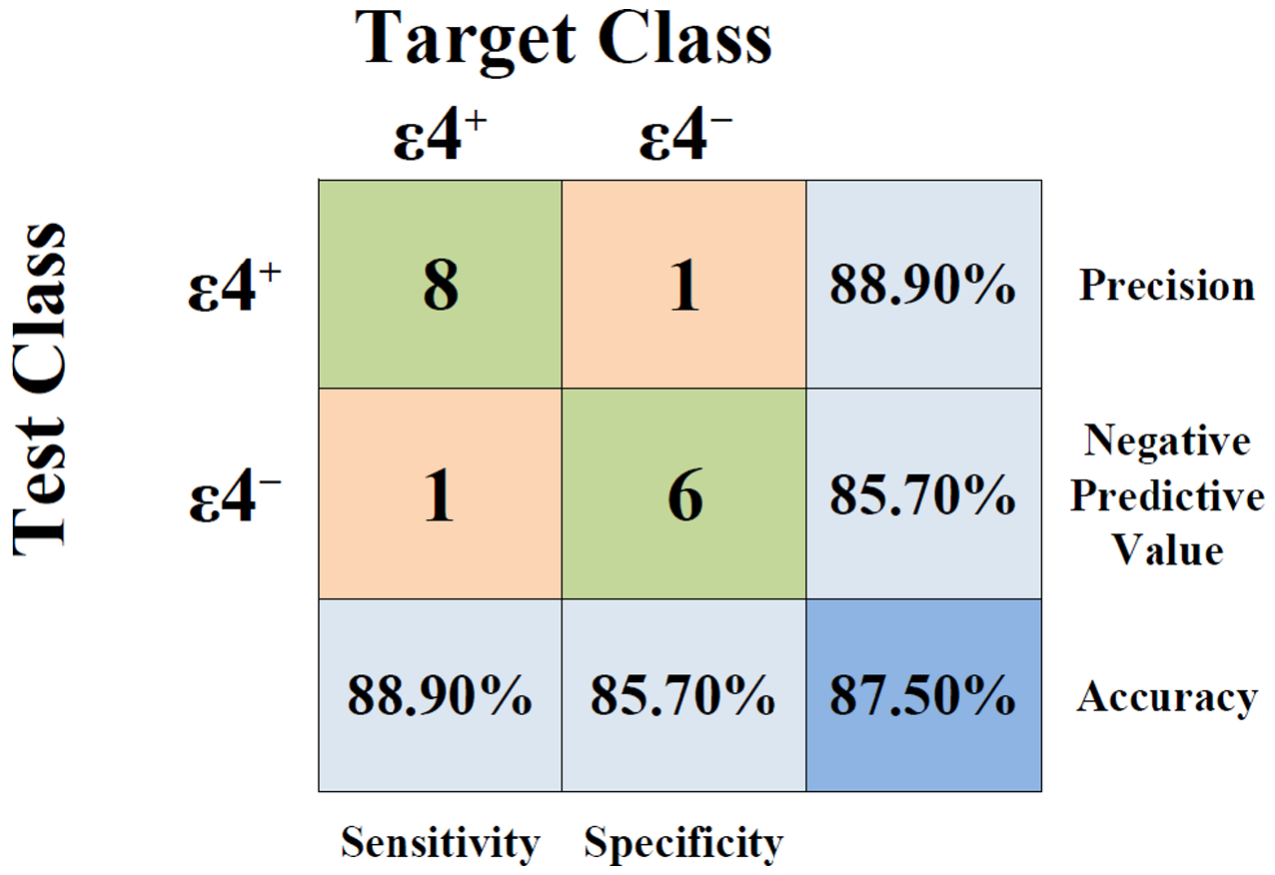
Confusion matrix for binary classification of APOE-ε4 carrier status (ε4^+^ vs. ε4^−^), with corresponding performance metrics including sensitivity, specificity, precision, negative predictive value, and accuracy.

The classification accuracy of lip movement features during the color-word interference speech task was evaluated across two genetically distinct groups (ε4^+^ and ε4^−^). We used an SVM with a degree-2 polynomial kernel and leave-one-out cross-validation as a classification model. Among the 15 extracted parameters, the combination of lip movement duration during the pre-interference segment, average movement speed during the interference segment, and the ∆_During-Pre_ difference in movement range yielded the highest classification accuracy. The best classification performance demonstrated an accuracy of 87.50%, with additional performance metrics including a precision of 88.90%, a sensitivity of 88.90%, and a specificity of 85.70% (Figure 2).

## Discussion

The present study examined the impact of APOE-ε4 genotype on lip movement kinematics during a color-word interference task, focusing on how mild cognitive interference modulates speech motor control. Descriptive analyses revealed moderate effect sizes for several kinematic features, even though group differences did not reach statistical significance. This pattern suggests that speech motor responses under cognitive load may capture early neuromotor vulnerability in APOE-ε4 carriers. The classification results offer preliminary, hypothesis-generating evidence that kinematic speech responses to cognitive interference could serve as early indicators of APOE-ε4-associated risk for AD, even in cognitively normal older adults.

It is important to note, however, that the gender imbalance observed in our sample (higher proportion of females in the ε4^+^ group) represents a critical confound; the ε4^+^ group included a higher proportion of females (66.67%) than the ε4^−^ group (28.57%). Sex has been shown to significantly modulate AD risk, with women exhibiting disproportionately higher AD incidence, faster neurodegenerative decline, and distinct neurocognitive resilience profiles in preclinical and prodromal stages compared to men (57–61). Women who carry the APOE-ε4 allele, in particular, are at greatest risk, showing accelerated progression, greater biomarker burden, and more pronounced motor and cognitive deficits relative to male carriers (57–59). Additionally, recent evidence indicates that sex differences influence facial and speech-related neuromuscular activity in APOE-ε4 carriers, with female carriers showing reduced and male carriers showing increased functional connectivity during speech (51), highlighting sex as a critical factor that may confound genotype effects when interpreting speech-based biomarkers for Alzheimer’s risk. Consequently, the trends reported here may reflect a combination of APOE-ε4-related vulnerability and sex-specific influences. This limitation restricts the interpretability of our results and highlights the urgent need for larger, sex-balanced samples allowing stratified analyses of male and female carriers.

Due to the small sample size and exhaustive feature search, the current findings should be interpreted as preliminary and require replication in larger, independent datasets, ideally with explicit adjustment or stratification for sex differences. Future work should systematically account for sex as a biological variable to enhance the validity and generalizability of speech-based biomarkers for AD risk.

For classification, the final feature set was selected from all possible combinations based solely on cross-validated accuracy. The model achieved a classification accuracy of 87.5% in distinguishing between APOE-ε4 carriers and non-carriers using three kinematic speech features: lip movement duration during the pre-interference segment, average lip movement speed during interference, and the change in lip movement range from pre- to during-interference. While this exhaustive approach maximizes the chance of identifying discriminative features, it also increases the risk for overfitting in small samples. Accordingly, the identified features should be considered as candidate, exploratory markers rather than definitive indicators of APOE-ε4 status.

### Speech motor adjustments reveal cognitive-motor vulnerability in APOE-ε4 carriers

Altered articulatory movement duration observed during the pre-interference segment reflects anticipatory motor adjustments and resource allocation in preparation for upcoming cognitive interference. This has been attributed to proactive modulation of speech motor planning, where speakers change (i.e., typically slow down) articulatory movements to buffer against predicted instability and to facilitate error monitoring as cognitive demands increase (62, 63). Predictive feedforward mechanisms within the motor system further contribute by prolonging movement duration to accommodate potential error margins under cognitive stress (64). Consistent with this interpretation, our descriptive data showed that pre-interference lip movement duration was moderately shorter in ε4^+^ individuals (*d* = −0.53), suggesting altered anticipatory motor strategies in this group. The inclusion of this feature in the final classification model underscores the potential diagnostic utility of pre-task articulatory dynamics in identifying APOE-ε4 status.

Similarly, prior research suggests that individuals under cognitive interference often adopt more conservative articulation strategies, such as slower and larger articulatory excursions, to maintain intelligibility and motor stability (53, 65, 66). In our study, the ∆_During-Pre_ difference in movement range emerged as a key feature, suggesting that changes in articulatory flexibility under cognitive load are informative for group classification. Although the machine learning model does not directly quantify the direction of group-level changes, our descriptive analysis revealed a smaller absolute ∆_During-Pre_ difference in lip movement range in the ε4^+^ group (0.205 mm^3^) compared to the ε4^−^ group (0.609 mm^3^), with a moderate effect size (*d* = −0.371). This reduced dynamic modulation may indicate diminished neuromotor adaptability to cognitive demands in ε4^+^ individuals.

Altered articulatory speed during the interference condition of a cognitive-motor task has been linked to increased susceptibility to cognitive-motor conflict, reflecting difficulty in maintaining speech precision while resolving competing demands. Prior research suggests that when cognitive control resources are heavily taxed, neural resources are reallocated from motor execution to processes such as conflict monitoring and inhibition, resulting in slower and less precise articulatory movements (67–70). These effects are especially pronounced in at-risk populations, including older adults and individuals with executive dysfunction, who tend to exhibit greater slowing and variability in movement during cognitive interference (71), further highlighting the close interplay between executive function and speech motor control (67–70). This pattern aligns with findings from dual-task gait studies, where APOE-ε4 carriers show slower walking speeds and greater reductions in step length when simultaneously engaged in executive function tasks (72). Although these findings focus on gross motor behavior, they illustrate a broader compensatory mechanism in which motor systems prioritize stability, often through slower or more deliberate movements, under increased cognitive load. In the present study, average lip movement speed during the interference segment did not differ significantly between groups (*d* = −0.18), but it contributed substantially to classification accuracy, reinforcing its relevance as a speech timing feature linked to cognitive load sensitivity.

Given that the two groups were matched by age, it is unlikely that the observed differences in speech kinematics are attributable to aging effects. However, the interaction between sex and APOE-ε4 status (as discussed above) may still substantially influence these kinematic outcomes. The trends observed in this study may therefore reflect not only genotype-related vulnerability, but also distinct sex-specific patterns in motor and cognitive adaptation that warrant further investigation. Future research should aim to disentangle the independent and interactive effects of genotype and sex on anticipatory and adaptive speech motor strategies.

### Potential clinical utility of speech-based biomarkers for APOE-ε4

These findings suggest the hypothesis that subtle speech motor differences in cognitively intact APOE-ε4 carriers may be detectable via articulatory kinematic measures, particularly under mild cognitive load. The model’s cross-validated classification accuracy of 87.5%, with corresponding precision (88.9%), sensitivity (88.9%), and specificity (85.7%), indicates that speech-derived features could potentially identify ε4^+^ individuals. Importantly, these metrics reflect the methodological approach in a limited dataset and must be interpreted as exploratory rather than confirming clinical utility.

Unlike traditional neuropsychological assessments that focus on cognitive decline, speech kinematics offer a behaviorally grounded, low-burden, and non-invasive window into early neuromotor changes, especially those affecting articulatory timing and precision. These features show promise as early indicators of genetic risk and may augment existing multimodal approaches to stratification, screening, and individualized risk monitoring. While speech kinematics show promise as a low-burden and scalable approach for risk stratification, the present study is underpowered to make clinical claims. Establishing clinical utility will require: (1) replication in larger and demographically diverse samples, (2) independent validation cohorts, (3) longitudinal tracking to determine predictive value for cognitive decline, and (4) comparison with established diagnostic methods and biomarkers. Until such work is completed, the present findings should be viewed as preliminary and hypothesis-generating.

Importantly, our results suggest that APOE-ε4 carriers with intact cognition may already exhibit early motor abnormalities, reinforcing the idea that motor deficits can precede overt cognitive decline. This aligns with previous research showing that individuals with the APOE-ε4 allele experience accelerated motor decline independent of detectable cognitive impairment (6, 25). The underlying mechanisms likely involve multiple neurobiological pathways. APOE-ε4 is strongly associated with increased amyloid-β deposition and tau pathology, which disrupt neural circuits critical for motor control (73–75). Additionally, synaptic loss and dysfunction in motor regions may impair the fine motor coordination necessary for speech production (73, 75). Neuroinflammation and cerebrovascular dysfunction, both characteristic features of APOE-ε4 carriers, could further contribute to early motor deficits (76–78). While our findings provide indirect behavioral evidence of such disruptions, future work integrating speech kinematics with imaging and molecular markers will be necessary to clarify the mechanistic basis of these early articulatory changes.

### Study limitations

Several limitations of this study should be acknowledged. First, the small sample size limits statistical power and reduces the ability to detect significant group differences or reliably assess the directionality of changes in individual kinematic features. This also increases the risk of overfitting in the machine learning model, restricts the broader interpretation of clinical implications, and limits generalizability. Therefore, the results should be viewed as hypothesis-generating that require replication and validation in larger, independent samples. Second, the cross-sectional design precludes evaluation of whether speech kinematic differences in APOE-ε4 carriers evolve longitudinally or predict future cognitive decline. Third, the absence of neuroimaging or other biological markers restricts mechanistic interpretation, making it difficult to determine whether the observed speech motor changes are linked to specific brain network alterations associated with APOE-ε4. Fourth, the lack of an MCI comparison group limits the ability to differentiate whether the articulatory changes observed are specific to preclinical AD risk or reflect broader patterns of cognitive aging. The unbalanced gender distribution within the ε4^+^ group represents a major confound. Given well-documented sex differences in APOE-ε4 effects, this imbalance reduces both generalizability and the ability to clarify potential sex contributions to speech kinematic changes. Our results may therefore reflect a combination of genotype and sex-related effects. Future studies should ensure sex-balanced recruitment and incorporate stratified analyses to disentangle sex-specific influences on speech motor outcomes in APOE-ε4 carriers. Finally, although the two groups were age-matched in our study, minimizing confounding due to general age-related motor changes, the observed differences in lip kinematics could nonetheless reflect broader motor control variability rather than AD-specific neural pathways. However, without corroborating neuroimaging or biomarker data, our findings should be interpreted as preliminary behavioral trends. Future research should incorporate multimodal designs integrating speech kinematics, neuroimaging, and fluid biomarkers to clarify the neurobiological underpinnings of these effects.

## Conclusion

This study indicates possible alterations in speech motor control among APOE-ε4 carriers, particularly under conditions of cognitive-linguistic interference during a color-word interference task. Machine learning analysis identified a combination of temporal and spatial speech kinematic features that accurately classified ε4^+^ individuals in this sample with 87.50% accuracy. These findings suggest that subtle disruptions in articulatory timing and flexibility may emerge in ε4^+^ carriers, potentially reflecting early neural vulnerabilities in motor adaptability, inhibitory control, and cognitive-motor integration. However, given the small sample size and cross-sectional design, these group-level trends should be interpreted as preliminary and hypothesis-generating rather than conclusive.

Future research should investigate whether these speech motor signatures intensify over time, track with cognitive decline, and improve the early detection of neurodegenerative risk. Incorporating speech kinematic assessments into precision-medicine frameworks may eventually enhance individualized risk profiling, especially if combined with genetic and fluid biomarkers. Nevertheless, the application of speech-based measures as scalable and non-invasive biomarkers for early detection and longitudinal monitoring in at-risk populations remains at an early, proof-of-concept stage. Establishing their reliability, sensitivity, and clinical utility will require validation in larger and more diverse samples, independent cohorts, and through direct comparison to established diagnostic methods and biomarkers.

## Data Availability

A de-identified dataset comprising the extracted kinematic features that support the findings of this study will be available from the corresponding author upon request.

## References

[1] Farrer LA. Effects of age, sex, and ethnicity on the association between apolipoprotein E genotype and Alzheimer disease: a meta-analysis. JAMA. (1997) 278:1349. doi: 10.1001/jama.1997.03550160069041, 9343467

[2] Corder EH Saunders AM Strittmatter WJ Schmechel DE Gaskell PC Small GW . Gene dose of apolipoprotein E type 4 allele and the risk of Alzheimer’s disease in late onset families. Science. (1993) 261:921–3. doi: 10.1126/science.8346443, 8346443

[3] Kim J Basak JM Holtzman DM. The role of apolipoprotein E in Alzheimer’s disease. Neuron. (2009) 63:287–303. doi: 10.1016/j.neuron.2009.06.026, 19679070 PMC3044446

[4] Sharad S Kapoor M Bala K. Apo E genotypes: risk factor for Alzheimer’s disease. J Indian Acad Clin Med. (2006) 7:118–22.

[5] Belloy ME Andrews SJ Le Guen Y Cuccaro M Farrer LA Napolioni V . APOE genotype and Alzheimer disease risk across age, sex, and population ancestry. JAMA Neurol. (2023) 80:1284–94. doi: 10.1001/jamaneurol.2023.3599, 37930705 PMC10628838

[6] Buchman AS Boyle PA Wilson RS Beck TL Kelly JF Bennett DA. Apolipoprotein E e4 allele is associated with more rapid motor decline in older persons. Alzheimer Dis Assoc Disord. (2009) 23:63–9. doi: 10.1097/WAD.0b013e31818877b5, 19266700 PMC2662708

[7] Sun J Zhu Z Chen K Wei D Li X Li H . APOE ε4 allele accelerates age-related multi-cognitive decline and white matter damage in non-demented elderly. Aging. (2020) 12:12019–31. doi: 10.18632/aging.103367, 32572010 PMC7343443

[8] Sakurai R Watanabe Y Osuka Y Taniguchi Y Kawai H Kim H . Overlap between apolipoprotein Eε4 allele and slowing gait results in cognitive impairment. Front Aging Neurosci. (2019) 11:247. doi: 10.3389/fnagi.2019.00247, 31572165 PMC6753959

[9] Ali JI Smart CM Gawryluk JR. Subjective cognitive decline and APOE ɛ4: a systematic review. J Alzheimers Dis. (2018) 65:303–20. doi: 10.3233/JAD-180248, 30040718

[10] Williams OA An Y Armstrong NM Shafer AT Helphrey J Kitner-Triolo M . Apolipoprotein E ε4 allele effects on longitudinal cognitive trajectories are sex and age dependent. Alzheimers Dement. (2019) 15:1558–67. doi: 10.1016/j.jalz.2019.07.011, 31561966 PMC7561018

[11] Ihle A Bunce D Kliegel M. APOE ε4 and cognitive function in early life: a meta-analysis. Neuropsychology. (2012) 26:267–77. doi: 10.1037/a0026769, 22329498

[12] Marioni RE Campbell A Scotland G Hayward C Porteous DJ Deary IJ. Differential effects of the APOE e4 allele on different domains of cognitive ability across the life-course. Eur J Hum Genet. (2016) 24:919–23. doi: 10.1038/ejhg.2015.210, 26395552 PMC4705436

[13] Du J Liu Z Hanford LC Anderson KM Feng J Ge T . (2021). Exploration of Alzheimer’s disease MRI biomarkers using APOE4 carrier status in the UK Biobank. *medRxiv*. Available online at: 10.1101/2021.09.09.21263324. [Epub ahead of preprint]

[14] O’Dwyer L Lamberton F Matura S Tanner C Scheibe M Miller J . Reduced hippocampal volume in healthy young ApoE4 carriers: an MRI study. PLoS One. (2012) 7:e48895. doi: 10.1371/journal.pone.0048895 23152815 PMC3494711

[15] Pievani M Galluzzi S Thompson PM Rasser PE Bonetti M Frisoni GB. APOE4 is associated with greater atrophy of the hippocampal formation in Alzheimer’s disease. NeuroImage. (2011) 55:909–19. doi: 10.1016/j.neuroimage.2010.12.081, 21224004

[16] Quevenco FC van Bergen JM Treyer V Studer ST Kagerer SM Meyer R . Functional brain network connectivity patterns associated with Normal cognition at old-age, local β-amyloid, tau, and APOE4. Front Aging Neurosci. (2020) 12:46. doi: 10.3389/fnagi.2020.00046, 32210782 PMC7075450

[17] Brown JA Terashima KH Burggren AC Ercoli LM Miller KJ Small GW . Brain network local interconnectivity loss in aging APOE-4 allele carriers. Proc Natl Acad Sci USA. (2011) 108:20760–5. doi: 10.1073/pnas.1109038108, 22106308 PMC3251140

[18] Luo Y Tan L Therriault J Zhang H Gao Y The Alzheimer’s Disease Neuroimaging Initiative. The role of apolipoprotein E ε4 in early and late mild cognitive impairment. Eur Neurol. (2021) 84:472–80. doi: 10.1159/000516774, 34340229

[19] Bailey M Ilchovska ZG Hosseini AA Jung J. Impact of apolipoprotein E ε4 in Alzheimer’s disease: a meta-analysis of voxel-based morphometry studies. J Clin Neurol. (2024) 20:469–77. doi: 10.3988/jcn.2024.0176, 39227329 PMC11372214

[20] Qian J Betensky RA Hyman BT Serrano-Pozo A. Association of APOE genotype with heterogeneity of cognitive decline rate in Alzheimer disease. Neurology. (2021) 96:e2414–28. doi: 10.1212/WNL.0000000000011883, 33771840 PMC8166439

[21] Bonham LW Geier EG Fan CC Leong JK Besser L Kukull WA . Age-dependent effects of APOE ε4 in preclinical Alzheimer’s disease. Ann Clin Transl Neurol. (2016) 3:668–77. doi: 10.1002/acn3.333, 27648456 PMC5018579

[22] Morris R Martini DN Kelly VE Smulders K Ramsey K Hiller A . Gait and balance in apolipoprotein Ɛ4 allele carriers in older adults and Parkinson’s disease. Clin Park Relat Disord. (2023) 9:100201. doi: 10.1016/j.prdoa.2023.100201, 37252677 PMC10209874

[23] Sakurai R Montero-Odasso M. Apolipoprotein E4 allele and gait performance in mild cognitive impairment: results from the gait and brain study. J Gerontol A. (2017) 72:1676–82. doi: 10.1093/gerona/glx075, 28482102 PMC5861852

[24] Pratt J Dalla Via J Sale C Gebre AK Stephan BCM Laws S . Apolipoprotein ɛ4 is associated with increased risk of fall- and fracture-related hospitalization: the Perth longitudinal study of ageing women. J Gerontol A Biol Sci Med Sci. (2024) 79:glae134. doi: 10.1093/gerona/glae134, 38766839 PMC11212482

[25] Skoog I Hörder H Frändin K Johansson L Östling S Blennow K . Association between APOE genotype and change in physical function in a population-based Swedish cohort of older individuals followed over four years. Front Aging Neurosci. (2016) 8:225. doi: 10.3389/fnagi.2016.00225, 27757080 PMC5047916

[26] Allard JS Ntekim O Johnson SP Ngwa JS Bond V Pinder D . APOEε4 impacts up-regulation of brain-derived neurotrophic factor after a six-month stretch and aerobic exercise intervention in mild cognitively impaired elderly African Americans: a pilot study. Exp Gerontol. (2017) 87:129–36. doi: 10.1016/j.exger.2016.11.001, 27864047 PMC5193139

[27] Palmer JM Huentelman M Ryan L. More than just risk for Alzheimer’s disease: APOE ε4’s impact on the aging brain. Trends Neurosci. (2023) 46:750–63. doi: 10.1016/j.tins.2023.06.003, 37460334

[28] Wolk DA Dickerson BC Weiner M Aiello M Aisen P Albert MS . Apolipoprotein E (APOE) genotype has dissociable effects on memory and attentional–executive network function in Alzheimer’s disease. Proc Natl Acad Sci USA. (2010) 107:10256–61. doi: 10.1073/pnas.1001412107, 20479234 PMC2890481

[29] Wang Z Dai Z Shu H Liao X Yue C Liu D . APOE genotype effects on intrinsic brain network connectivity in patients with amnestic mild cognitive impairment. Sci Rep. (2017) 7:397. doi: 10.1038/s41598-017-00432-0, 28341847 PMC5428452

[30] Scarpina F Tagini S. The Stroop color and word test. Front Psychol. (2017) 8:557. doi: 10.3389/fpsyg.2017.00557, 28446889 PMC5388755

[31] Stroop JR. Studies of interference in serial verbal reactions. J Exp Psychol. (1935) 18:643–62. doi: 10.1037/h0054651

[32] Spieler DH Balota DA Faust ME. Stroop performance in healthy younger and older adults and in individuals with dementia of the Alzheimer’s type. J Exp Psychol Hum Percept Perform. (1996) 22:461–79. doi: 10.1037/0096-1523.22.2.461, 8934854

[33] Hutchison KA Balota DA Duchek JM. The utility of Stroop task switching as a marker for early-stage Alzheimer’s disease. Psychol Aging. (2010) 25:545–59. doi: 10.1037/a0018498, 20853964 PMC2946107

[34] Fan C Li H Chen K Yang G Xie H Li H . Brain compensatory activation during Stroop task in patients with mild cognitive impairment: a functional near-infrared spectroscopy study. Front Aging Neurosci. (2025) 17:1470747. doi: 10.3389/fnagi.2025.1470747, 39990105 PMC11842388

[35] Guarino A Favieri F Boncompagni I Agostini F Cantone M Casagrande M. Executive functions in Alzheimer disease: a systematic review. Front Aging Neurosci. (2019) 10:437. doi: 10.3389/fnagi.2018.00437, 30697157 PMC6341024

[36] Balota DA Tse CS Hutchison KA Spieler DH Duchek JM Morris JC. Predicting conversion to dementia of the Alzheimer’s type in a healthy control sample: the power of errors in Stroop color naming. Psychol Aging. (2010) 25:208–18. doi: 10.1037/a0017474, 20230140 PMC2886285

[37] Silva PCD De Oliveira LLV Teixeira RLP Brito MLDA Filippe ARTM. Executive functions in Alzheimer’s disease: a systematic review. J Alzheimers Dis Rep. (2022) 6:81–99. doi: 10.3233/ADR-210059

[38] Ben-David BM Tewari A Shakuf V Van Lieshout PHHM. Stroop effects in Alzheimer’s disease: selective attention speed of processing, or color-naming? A meta-analysis. J Alzheimers Dis. (2014) 38:923–38. doi: 10.3233/JAD-131244 24100125

[39] Patten RV Fagan AM Kaufman DAS. Differential cued-Stroop performance in cognitively asymptomatic older adults with biomarker-identified risk for Alzheimer’s disease: a pilot study. Curr Alzheimer Res. (2018) 15:820–7. doi: 10.2174/1567205015666180404170359, 29623843

[40] Duchek JM Balota DA Thomas JB Snyder AZ Rich P Benzinger TL . Relationship between Stroop performance and resting state functional connectivity in cognitively normal older adults. Neuropsychology. (2013) 27:516–28. doi: 10.1037/a0033402, 24040929 PMC3837537

[41] Bejanin A Schonhaut DR La Joie R Kramer JH Baker SL Sosa N . Tau pathology and neurodegeneration contribute to cognitive impairment in Alzheimer’s disease. Brain. (2017) 140:3286–300. doi: 10.1093/brain/awx243, 29053874 PMC5841139

[42] Kaiser A Kuhlmann BG Bosnjak M. A meta-analysis of inhibitory-control deficits in patients diagnosed with Alzheimer’s dementia. Neuropsychology. (2018) 32:615–33. doi: 10.1037/neu0000460, 29745708

[43] Saeedi S Hetjens S Grimm MOW Barsties V Latoszek B. Acoustic speech analysis in Alzheimer’s disease: a systematic review and meta-analysis. J Prev Alzheimers Dis. (2024) 11:1789–97. doi: 10.14283/jpad.2024.132 39559890 PMC11573841

[44] Martínez-Nicolás I Llorente TE Martínez-Sánchez F Meilán JJG. Ten years of research on automatic voice and speech analysis of people with Alzheimer’s disease and mild cognitive impairment: a systematic review article. Front Psychol. (2021) 12:620251. doi: 10.3389/fpsyg.2021.620251, 33833713 PMC8021952

[45] Vigo I Coelho L Reis S. Speech- and language-based classification of Alzheimer’s disease: a systematic review. Bioengineering. (2022) 9:27. doi: 10.3390/bioengineering9010027, 35049736 PMC8772820

[46] Szatloczki G Hoffmann I Vincze V Kalman J Pakaski M. Speaking in Alzheimer’s disease, is that an early sign? Importance of changes in language abilities in Alzheimer’s disease. Front Aging Neurosci. (2015) 7:195. doi: 10.3389/fnagi.2015.00195, 26539107 PMC4611852

[47] Shellikeri S Green JR Kulkarni M Rong P Martino R Zinman L . Speech movement measures as markers of bulbar disease in amyotrophic lateral sclerosis. J Speech Lang Hear Res. (2016) 59:887–99. doi: 10.1044/2016_JSLHR-S-15-0238, 27679842 PMC5345561

[48] Eshghi M Stipancic KL Mefferd A Rong P Berry JD Yunusova Y . Assessing oromotor capacity in ALS: the effect of a fixed-target task on lip biomechanics. Front Neurol. (2019) 10:1288. doi: 10.3389/fneur.2019.01288, 31866935 PMC6906194

[49] Eshghi M Rong P Mefferd AS Stipancic KL Yunusova Y Green JR. (2019). Reduced task adaptation in alternating motion rate tasks as an early marker of bulbar involvement in amyotrophic lateral sclerosis. INTERSPEECH. 4524–4528

[50] Rong P Yunusova Y Eshghi M Rowe HP Green JR. A speech measure for early stratification of fast and slow progressors of bulbar amyotrophic lateral sclerosis: lip movement jitter. Amyotroph Lateral Scler Frontotemporal Degener. (2020) 21:34–41. doi: 10.1080/21678421.2019.1681454, 31694409 PMC7060806

[51] Eshghi M Rong P Dadgostar M Shin H Richburg BD Barnett NV . (2025) APOE- ε4 modulates facial neuromuscular activity in nondemented adults: toward sensitive speech-based diagnostics for Alzheimer’s disease. *medRxiv*. Available online at: 10.1101/2025.04.29.25326665. [Epub ahead of preprint]

[52] Ishihara S. Tests for colour-blindness. Tokyo: Kanehara Shuppan (1972).

[53] MacPherson MK. Cognitive load affects speech motor performance differently in older and younger adults. J Speech Lang Hear Res. (2019) 62:1258–77. doi: 10.1044/2018_JSLHR-S-17-0222, 31051090

[54] Belleville S Chertkow H Gauthier S. Working memory and control of attention in persons with Alzheimer’s disease and mild cognitive impairment. Neuropsychology. (2007) 21:458–69. doi: 10.1037/0894-4105.21.4.458, 17605579

[55] Luo J Zeng Y Ye Y Xiao Y Xie Q Zhang J . Attention performance and altered amplitude of low-frequency fluctuations in the attention network of patients with MCI: a resting-state functional MRI study. J Integr Neurosci. (2025) 24:36464. doi: 10.31083/JIN36464, 40302268

[56] Green JR Wang J Wilson DL. (2013). SMASH: a tool for articulatory data processing and analysis. INTERSPEECH. 1331–1335

[57] Holland D Desikan RS Dale AM McEvoy LK Alzheimer’s Disease Neuroimaging Initiative. Higher rates of decline for women and apolipoprotein E epsilon4 carriers. AJNR Am J Neuroradiol. (2013) 34:2287–93. doi: 10.3174/ajnr.A3601, 23828104 PMC3894062

[58] Valencia-Olvera AC Maldonado Weng J Christensen A LaDu MJ Pike CJ. Role of estrogen in women’s Alzheimer’s disease risk as modified by APOE. J Neuroendocrinol. (2023) 35:e13209. doi: 10.1111/jne.13209, 36420620 PMC10049970

[59] O’Neal MA. Women and the risk of Alzheimer’s disease. Front Glob Womens Health. (2024) 4:1324522. doi: 10.3389/fgwh.2023.1324522, 38250748 PMC10796575

[60] Bourzac K. Why women experience Alzheimer’s disease differently from men. Nature. (2025) 640:S14–7. doi: 10.1038/d41586-025-01106-y, 40240843

[61] Eissman JM Dumitrescu L Mahoney ER Smith AN Mukherjee S Lee ML . Sex differences in the genetic architecture of cognitive resilience to Alzheimer’s disease. Brain. (2022) 145:2541–54. doi: 10.1093/brain/awac177, 35552371 PMC9337804

[62] Tilsen S. Selection and coordination: the articulatory basis for the emergence of phonological structure. J Phon. (2016) 55:53–77. doi: 10.1016/j.wocn.2015.11.005

[63] Guenther FH. Neural control of speech. Cambridge, MA: The MIT Press (2016).

[64] Houde JF Nagarajan SS. Speech production as state feedback control. Front Hum Neurosci. (2011) 5:82. doi: 10.3389/fnhum.2011.00082, 22046152 PMC3200525

[65] Rodgers JD Tjaden K Feenaughty L Weinstock-Guttman B Benedict RHB. Influence of cognitive function on speech and articulation rate in multiple sclerosis. J Int Neuropsychol Soc. (2013) 19:173–80. doi: 10.1017/S1355617712001166, 23058309 PMC5564302

[66] Hammons SG. Articulation as a predictor of cognitive decline In: Senior theses. Columbia, SC: University of South Carolina (2025)

[67] Kemper S Herman RE Nartowicz J. Different effects of dual task demands on the speech of young and older adults. Aging Neuropsychol Cognit. (2005) 12:340–58. doi: 10.1080/138255890968466, 16557294 PMC1410812

[68] Kemper S Schmalzried R Hoffman L Herman R. Aging and the vulnerability of speech to dual task demands. Psychol Aging. (2010) 25:949–62. doi: 10.1037/a0020000, 21186917 PMC3050491

[69] Pernon M Fournet M Fougeron C Laganaro M. (2019). Dual-task effects on speech and non-verbal tasks according to tasks properties. 19th International Congress of Phonetic Sciences.

[70] Kemper S Hoffman L Schmalzried R Herman R Kieweg D. Tracking talking: dual task costs of planning and producing speech for young versus older adults. Aging Neuropsychol Cognit. (2011) 18:257–79. doi: 10.1080/13825585.2010.527317, 21140310 PMC3091967

[71] Plummer-D’Amato P Altmann LJP Reilly K. Dual-task effects of spontaneous speech and executive function on gait in aging: exaggerated effects in slow walkers. Gait Posture. (2011) 33:233–7. doi: 10.1016/j.gaitpost.2010.11.011, 21193313

[72] Whitson HE Potter GG Feld JA Plassman BL Reynolds K Sloane R . Dual-task gait and Alzheimer’s disease genetic risk in cognitively normal adults: a pilot study. J Alzheimer's Dis. (2018) 64:1137–48. doi: 10.3233/JAD-180016, 30010120 PMC6500574

[73] Liu CC Kanekiyo T Xu H Bu G. Apolipoprotein E and Alzheimer disease: risk, mechanisms and therapy. Nat Rev Neurol. (2013) 9:106–18. doi: 10.1038/nrneurol.2012.263, 23296339 PMC3726719

[74] Hampel H Hardy J Blennow K Chen C Perry G Kim SH . The amyloid-β pathway in Alzheimer’s disease. Mol Psychiatry. (2021) 26:5481–503. doi: 10.1038/s41380-021-01249-0, 34456336 PMC8758495

[75] Baek MS Cho H Lee HS Lee JH Ryu YH Lyoo CH. Effect of APOE ε4 genotype on amyloid-β and tau accumulation in Alzheimer’s disease. Alzheimer's Res Ther. (2020) 12:140. doi: 10.1186/s13195-020-00710-6, 33129364 PMC7603688

[76] Zlokovic BV. Neurovascular pathways to neurodegeneration in Alzheimer’s disease and other disorders. Nat Rev Neurosci. (2011) 12:723–38. doi: 10.1038/nrn3114, 22048062 PMC4036520

[77] Montagne A Nation DA Sagare AP Barisano G Sweeney MD Chakhoyan A . APOE4 leads to blood–brain barrier dysfunction predicting cognitive decline. Nature. (2020) 581:71–6. doi: 10.1038/s41586-020-2247-3, 32376954 PMC7250000

[78] Foley KE Wilcock DM. Three major effects of APOEε4 on aβ immunotherapy induced ARIA. Front Aging Neurosci. (2024) 16:1412006. doi: 10.3389/fnagi.2024.1412006, 38756535 PMC11096466

